# Herpes Simplex Virus Type 1 Neuronal Infection Perturbs Golgi Apparatus Integrity through Activation of Src Tyrosine Kinase and Dyn-2 GTPase

**DOI:** 10.3389/fcimb.2017.00371

**Published:** 2017-08-22

**Authors:** Carolina Martin, Luis Leyton, Melissa Hott, Yennyfer Arancibia, Carlos Spichiger, Mark A. McNiven, Felipe A. Court, Margarita I. Concha, Patricia V. Burgos, Carola Otth

**Affiliations:** ^1^Faculty of Medicine, Institute of Clinical Microbiology, Universidad Austral de Chile Valdivia, Chile; ^2^Department of Biochemistry and Molecular Biology and the Center for Basic Research in Digestive Diseases, Mayo Clinic Rochester, MN, United States; ^3^Center for Integrative Biology, Faculty of Sciences, Universidad Mayor Santiago, Chile; ^4^Faculty of Sciences, Institute of Biochemistry and Microbiology, Universidad Austral de Chile Valdivia, Chile; ^5^Faculty of Medicine, Institute of Physiology, Universidad Austral de Chile Valdivia, Chile; ^6^Facultad de Ciencia y Facultad de Medicina, Centro de Biología Celular y Biomedicina, Universidad San Sebastián Santiago, Chile; ^7^Centro Interdisciplinario de Estudios del Sistema Nervioso (CISNe), Universidad Austral de Chile Valdivia, Chile

**Keywords:** HSV-1, Golgi fragmentation, neuronal dysfunction, Src, dynamin, neurodegeneration, vesicular trafficking

## Abstract

Herpes simplex virus type 1 (HSV-1) is a ubiquitous pathogen that establishes a latent persistent neuronal infection in humans. The pathogenic effects of repeated viral reactivation in infected neurons are still unknown. Several studies have reported that during HSV-1 epithelial infection, the virus could modulate diverse cell signaling pathways remodeling the Golgi apparatus (GA) membranes, but the molecular mechanisms implicated, and the functional consequences to neurons is currently unknown. Here we report that infection of primary neuronal cultures with HSV-1 triggers Src tyrosine kinase activation and subsequent phosphorylation of Dynamin 2 GTPase, two players with a role in GA integrity maintenance. Immunofluorescence analyses showed that HSV-1 productive neuronal infection caused a scattered and fragmented distribution of the GA through the cytoplasm, contrasting with the uniform perinuclear distribution pattern observed in control cells. In addition, transmission electron microscopy revealed swollen cisternae and disorganized stacks in HSV-1 infected neurons compared to control cells. Interestingly, PP2, a selective inhibitor for Src-family kinases markedly reduced these morphological alterations of the GA induced by HSV-1 infection strongly supporting the possible involvement of Src tyrosine kinase. Finally, we showed that HSV-1 tegument protein VP11/12 is necessary but not sufficient to induce Dyn2 phosphorylation. Altogether, these results show that HSV-1 neuronal infection triggers activation of Src tyrosine kinase, phosphorylation of Dynamin 2 GTPase, and perturbation of GA integrity. These findings suggest a possible neuropathogenic mechanism triggered by HSV-1 infection, which could involve dysfunction of the secretory system in neurons and central nervous system.

## Introduction

Herpes simplex virus type 1 (HSV-1) is a DNA enveloped virus, ubiquitous, neurotropic, and the most common pathogenic cause of sporadic acute encephalitis in humans (Whitley, 2006; Steiner, 2011). HSV-1 has the ability to establish and maintain a life-long latent infection in peripheral neurons with frequent reactivation episode, generally asymptomatic (Kastrukoff et al., 2012; Flowerdew et al., 2013; Tsalenchuck et al., 2014).

Several studies have suggested HSV-1 as an environmental risk factor for Alzheimer's disease (AD), in part because in herpes simplex encephalitis (HSE) the main regions affected are the temporal and frontal cortices and the hippocampus (Saldanha et al., 1986; Jamieson et al., 1992). In addition, HSV-1 DNA has been detected in the brain of normal aged individuals as well as in AD patients (Itzhaki et al., 2004; Wozniak et al., 2005).

The fact that the virus is present in the CNS of 70% of the population over 50 years old (Wozniak et al., 2005) suggests that recurrent reactivations in infected individuals could lead to neuronal alterations (for review see Itzhaki et al., 2004, 2016). However, the pathogenic mechanism of HSV-1 at the central nervous system (CNS), and the possibility of neuronal dysfunction are yet unknown (Mori, 2010; Roizman et al., 2013).

Many reports have demonstrated that viruses that establish persistent infections interact with members of the cellular Src family kinases (SFKs), which regulate several signaling pathways involved in cellular morphology, motility, proliferation, and survival (Miller et al., 1995; Collete and Olive, 1997; MacDonald et al., 2004; Pan et al., 2013; Szalmás et al., 2013; McCarthy et al., 2014). In this context, Liang and Roizman (2006) demonstrated that HSV-1 regulates the activity of members of the SFKs, specifically Src kinases, inducing optimal viral replication in infected epithelial cells. Later, it was discovered that activation of Src tyrosine kinase is triggered by phosphorylation of its residue Y424, whereas persistent activation affects integrity of the Golgi apparatus (GA) in a variety of cell types (Weller et al., 2010). One Src substrate implicated in this process is the large GTPase Dynamin 2 (Dyn2) (Weller et al., 2010). Activated Src triggers Dyn2 phosphorylation at two different positions, Y231 and Y597, modifications that positively regulate its GTPase activity (Cao et al., 2010). Dyn2 is a GTPase that controls the GA dynamics as well as *trans*-Golgi network (TGN) fragmentation during the secretory process in normal and neoplastic cells (Ishida et al., 2011). It also participates in synaptic vesicle recycling, post-synaptic receptor internalization, neurosecretion, and extension of neuronal processes in the nervous system (González-Jamett et al., 2014). Interestingly, the DynY231/597F tyrosine phospho-mutant reduced the fragmentation of the GA (Weller et al., 2010), altogether demonstrating that both, Src and Dyn2, regulate the integrity and function of the GA (Weller et al., 2010). Although, Dyn2 has several isoforms, the involvement of Dyn2 in neurological conditions points to a critical function of this isoform in the nervous system (González-Jamett et al., 2014). Interestingly, Campadelli et al. (1993) had previously reported that HSV-1 infection of Vero and Hep-2 cells causes fragmentation and dispersal of Golgi proteins and redistribution of glycoprotein and glycoplipids processed through the GA. These results were interpreted as processes required for late events of the viral reproductive cycle, characterized by maturation, processing, and sorting of virions (Campadelli et al., 1993). However, the potentially deleterious effects that structural and functional changes of the GA during HSV-1 neuronal infection could have on synaptic vesicle transport along axons have not been evaluated. In fact, previous studies have found that the fragmentation and dispersion of the GA precedes neuronal death triggered by excitotoxins, oxidative stress, and endoplasmic reticulum stress (Chiu et al., 2002; Lane et al., 2002; Machamer, 2003). These events have also been observed in several neurodegenerative diseases models (Stieber et al., 2004; Gonatas et al., 2006). Interestingly, previous studies in Jurkat T cells have shown that lymphocyte-specific Src family kinase Lck is activated during that HSV-1 infection (Zahariadis et al., 2008; Wagner and Smiley, 2009). In fact, the tegument protein VP11/12 has been identified as one specific substrate of Lck in T cells (Zahariadis et al., 2008; Wagner and Smiley, 2009). In this regard, the C-terminal region of VP11/12 contains tyrosine-based motifs predicted to bind the SH2 domains of SFKs (Strunk et al., 2013).

The aim of the present study was to evaluate whether HSV-1 neuronal infection triggers activation of Src tyrosine kinase resulting in alteration of the structure and integrity of GA and to study the possible involvement of the HSV-1 tegument protein VP11/12 in this process. Our results show that HSV-1 perturbs the GA in infected neurons, as demonstrated by immunofluorescence and transmission electron microscopy studies. Consistent with these findings, we show that HSV-1 neuronal infection activates Src tyrosine kinase and Dyn2 GTPase, two players functionally implicated in the maintenance of the GA integrity. Finally, we determined that VP11/12 is required for efficient HSV-1 replication in neurons, for Dyn2 activation and fragmentation of the GA during HSV-1 replicative life cycle. Therefore, we propose that HSV-1 neuronal infection have a profound impact in the neuronal secretory system explaining part of the deleterious effects of HSV-1 in central nervous system in humans.

## Materials and methods

### Biosafety methodology

Our laboratories have the necessary suitable procedures and biosafety equipment for biological elements and chemicals disposal, according to the Safety Manual of Procedures and Handling of Wastes from the Universidad Austral de Chile, and Bio-safety Regulations from CONICYT. Moreover, biosafety measures and containment barriers recommended by CONICYT and WHO for type II risk organisms are routinely used. The Laboratory of Molecular Virology is equipped with a level 2 biosafety chamber where we carried out HSV-1 propagation and infection experiments. All the activities were authorized and supervised by the principal investigator.

### Cell culture and viruses

Vero cells (American Type Culture Collection) were maintained in Dulbecco's modified Eagle's medium (DMEM) supplemented with 10% fetal bovine serum (Life Technology, Inc), 100 U/ml penicillin, and 100 μg/ml streptomycin in 5% CO_2_ and 95% air at 37°C.

Primary cortical neuronal culture was established with 17 days old mice embryos (E17) (Otth et al., 2007). The animals were sacrificed by lethal doses of intravenous sodium pentabarbitone (200 mg/kg of total weight). Death was confirmed observing cessation of heartbeat and respiration, and absence of reflexes, in agreement with international standards (http://www.lal.org.uk). Briefly, embryos (E17) were removed from mice and dissected cortex pairs. All the tissues were collected in a conical tube containing Hibernate-E complete medium (Gibco, NY, USA) supplemented with 200 mM glutamine and B-27 50X. The tissue was treated with 0.25% trypsin at 37°C for 5 min and then disaggregated by mechanical grinding with a sterile, fire-polished glass Pasteur pipette, in DMEM supplemented with 10% FBS. Cells were seeded onto coverslips or in 35-mm plastic dishes pre-coated with 10 μg/ml poly-lysine (mol. wt > 350 kDa; Sigma-Aldrich Corporation, St Louis, MO, USA). After 30 min in 5% CO_2_ and 95% air at 37°C, floating cells were removed and attached cells cultured for 7 days in Neurobasal Medium (Gibco, NY, USA) supplemented with B27 (Gibco, NY, USA), 100 U/ml penicillin, 100 μg/ml streptomycin, and 0.5 mM L-glutamine (Nalgene, Rochester, NY, USA).

HSV-1 (strain F) used in this study including HSV-1 wild type and HSV-1 ΔUL46 mutant (Zhang et al., 1991) were kindly supplied by Dr. Bernard Roizman, Northwestern University, Chicago, USA. All virus stocks were prepared and titrated from infected Vero cells (Ejercito et al., 1968; Zambrano et al., 2008).

### Infection protocols

For kinetics studies, infections with HSV-1 wild type or HSV-1 ΔUL46 mutant were carried out for 1 h in a DMEM containing 10% FBS, at multiplicity of infection (moi) of 10. Following infections, viruses were removed by washings, the medium was replaced by a serum-free medium consisting of Neurobasal medium supplemented with B27 1X and 0.5 mM L-glutamine, and the cells were further cultured for 4, 8, and 18 h post-infection (hpi). For the Src kinase inhibition experiment, cells were treated with the PP2 inhibitor (20 μM; Sigma-Aldrich Corporation St Louis, MO, USA) for 12 h before infection and maintained during infection with the virus.

### TCID50 assay protocol

To determine the amount of infectious viral particles, present in the cell extracts, we performed the TCID50 assay (Tissue Cultured Infective Dose). Vero cells were plated in a 96-well plate and grown, until they reached 80% of confluence. Serial dilutions of the virus sample were made (quadruplicate) and incubated with the Vero cells for 1 h at 37°C. Media were aspirated and fresh DMEM containing 5% FBS was added, incubating the plates at 37°C for 4 days. Subsequently, the medium was removed, and cells were incubated with 75% methanol for 15 min and stained with crystal violet for 1 min. The plates were washed with water and the number of wells in which the cell monolayer was detached from each dilution was counted. The titer of the virus stock was expressed as the TCID50, which was calculated using a statistical Excel program. The viral titer (PFU/ml) was achieved by multiplying the factor 0.69 TCID50/mL according to the Reed-Muench method (Reed and Muench, 1938).

### Fluorescence microscopy and quantitative analysis

Non-infected neurons (MOCK) and HSV-1 infected neurons, moi = 10, (treated or untreated with PP2 inhibitor) were fixed in 4% paraformaldehyde in PBS 1X for 30 min, washed in PBS 1X, and permeabilized in 0.2% Triton X-100 in PBS 1X for 5–10 min. Cells were incubated overnight at 4°C with the following primary antibodies diluted in PBS 1X. We used primary antibodies against the following proteins: HSV-1 ICP8 viral protein (sc-53329, clone 10A3, Santa Cruz), p-Src (sc-101802, Santa Cruz; recommended for the detection of mouse Src phosphorylated at Tyr424), Giantin (ab 248526, Abcam), and p-Dyn2 (Dyn2 phosphorylated at Tyr231; antibody donated by Dr. MacNiven, Mayo Clinic, Rochester-Minessota, USA). Finally, cells were incubated with the corresponding secondary antibodies conjugated with Alexa-488 or Alexa-594, and nuclei were stained with DAPI (Invitrogen). Fluorescence images were obtained using a Zeiss Axioskope A1 epifluorescence microscope (Carl Zeiss, Göttingen, Germany) with a digital video camera (Nikon DXM 1200). The images obtained (at least five microscopic fields per each sample) were processed with Adobe Photoshop 6.0. The phenotype of the GA was categorized according to normal or fragmented patterns observed by staining with the antibody against Giantin (a Golgi marker). The normal phenotype corresponded to homogenous staining clustered in the perinuclear region. The fragmented phenotype (FRAGM) corresponded to punctate staining scattered throughout the cytoplasm. The percentage of cells showing normal or fragmented distribution of Giantin was calculated from five digital images acquired for each condition. In addition, we measured the distance of Giantin positive structures to the nucleus as a parameter of GA dispersion. To this, immunofluorescence images (10 cells for each condition) obtained from infected neurons with either HSV-1 wild type or HSV-1ΔUL46 treated in the absence or presence of PP2 inhibitor were analyzed in each condition. Using Image J software (U.S. National Institutes of Health), each image was calibrated (pixels/μm), and the maximal distance of the Golgi marker with respect to the nucleus was measured in cells randomly selected. The data represent the average of maximal distance from the nucleus obtained from three independent experiments.

### Electron microscopy analysis

For Electron Microscopy analysis, we used neurons at a confluence of 2.1 × 10^6^ cells/cm^2^. Neurons were growth in Neurobasal media supplemented with B27, glutamine and 1% antibiotics. Once the time of infection was finished, the Neurobasal media was removed and each plate with cells was gently washed with PBS 1X. Then, neurons were fixed for 3 min at room temperature in 3% glutaraldehyde, 0.01% picric acid, and 50 mM cacodylate buffer, pH 7.4. Cells were collected in a 1.5 ml Eppendorf tube, and then centrifuged at 3,000–5,000 rpm for 3–5 min at room temperature until a pellet of cells appeared. The pellet was incubated in the same buffer with 1% OsO_4_ and then immersed in 2% uranyl acetate, dehydrated in a gradient of ethanol and acetone, and infiltrated in Epon resin (Ted Pella Inc.). Ultrathin sections were obtained using an ultramicrotome (Reichert). Thin 50–80 nm sections were obtained and mounted in copper grids and contrasted using 1% uranyl acetate and lead citrate. Observations of the grids were made using a Phillips Tecnai 12 (Eindhoven) at 80 kV and photographed using a Mega view G2 camera (Olympus) (Villegas et al., 2014). The Golgi morphology was analyzed in the sections obtained previously. The normal Golgi was defined as a highly ordered, pericentriolar, ribbon-like structure, and the fragmented Golgi was defined as scattered dots (not connected) in the perinuclear region, or multiple mini-Golgi (isolated dots) dissociated from the major GA. For the quantification of Golgi cisternae lumen width (μm) in mock- and HSV-1-infected cells, or in cells treated with the Src inhibitor PP2 (20 μM), we also quantified the maximum length of GA cisternae (designated as maximum luminal width) by measuring the cisternae diameters assuming circular structures. A number of 3–5 GA cisternae for each time were analyzed by EM. All images were analyzed using Image J software (Joshi et al., 2014).

### MTT assay

Cell viability was determined by the MTT-based colorimetric assay (3 - [4,5 – dimethyl thiazol -2 -yl] -2,5- diphenyl tetrazolium bromide), Sigma-Aldrich Corporation St Louis, MO, USA) modified by Liu and Schubert (1997). Neurons were cultured in 96-well plates until they reached 80% of confluence in a final volume of 100 μl of medium containing 10% FBS and without phenol red, and incubated at 37°C in 5% CO_2_. After kinetic infection, the mitochondrial activity was measured by the modified MTT assay (Mosmann, 1983). This assay involves determining mitochondrial dehydrogenase activity in intact cells by incubation for 4 h at 37°C with MTT (5 mg/ml MTT solution per well). After the incubation, the resulting complex was solubilized by the addition of an acidified ethanol solution, and the absorbance was read at 570 nm on a Microplate reader (Thermo, Multiskan MS), and the results were expressed as a percentage (%) of the control cells. The results were analyzed statistically using the non-parametric Student *t*-test.

### Western blotting

For protein analysis, neurons from different treatments were harvested and lysed in RIPA buffer 1X supplemented with protease and phosphatase inhibitors (Sigma-Aldrich Corporation St Louis, MO, USA) and the protein concentration was quantified with Micro BCA™ Protein Assay Kit (Thermo Fischer Scientific, Waltham, MA USA). Equal amounts of protein (20 μg per lane) were loaded and separated by gradient SDS-PAGE (8–20% polyacrylamide) and transferred to nitrocellulose membrane (Thermo Fischer Scientific, Waltham, MA USA). The membranes were incubated at room temperature in blocking solution (2% BSA in TBS-Tween), and then incubated overnight with primary antibodies diluted in TBS-1% BSA: anti-β-tubulin (Sigma-Aldrich Corporation St Louis, MO, USA ab6096), and p-Src (sc-101802), anti-Src (sc-5266), and anti-Dyn II (sc-166526) from Santa Cruz Biotechnology. Next were incubated with appropriate secondary antibodies (anti-rabbit and anti-mouse from Thermo Fischer Scientific, Waltham, MA USA) conjugated to peroxidase. The bands were detected using the enhanced chemiluminescence assay (SuperSignal West Pico Chemiluminiscent Substrate, Thermo Fischer Scientific, and Waltham, MA USA), and the CL-XPosure™ (X-Ray) films (Thermo Fischer Scientific, Waltham, MA USA). The films were scanned, and the resulting images were analyzed to determine the relative levels of each protein, using the Un-Scan-IT gel 6.1 software.

### Statistical analysis

All the results are representative of at least three independent experiments. Results were analyzed by One-Way or Two-Way ANOVA. The data were expressed as means ± standard deviations. The *p*-values were reported in each case; *p* < 0.05 was considered significant.

## Results

### HSV-1 neuronal infection triggers Src tyrosine kinase and dynamin 2 phosphorylation

**Figure 1:**
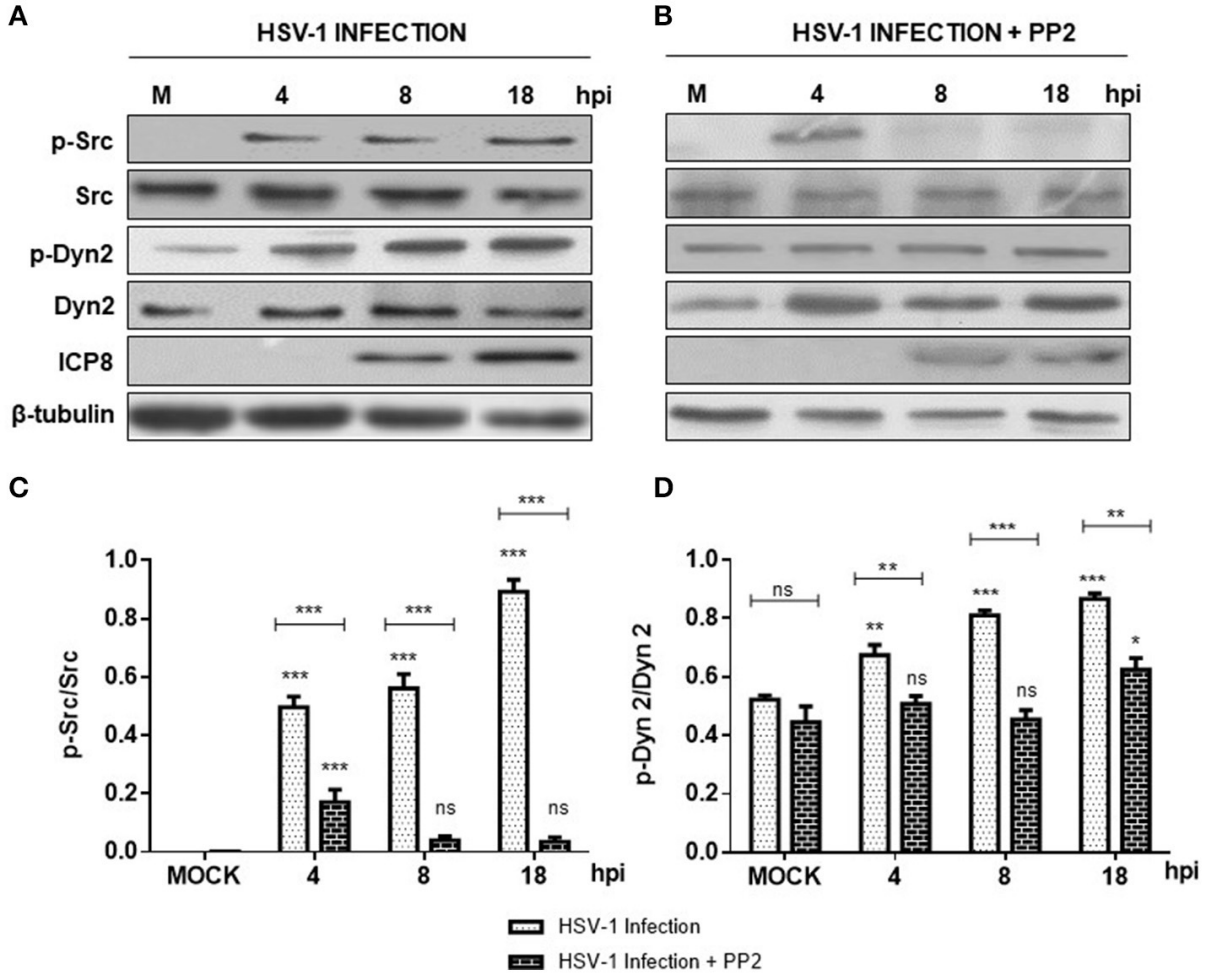
Src kinase and dynamin 2 activate during HSV-1 neuronal infection. Cortical primary neurons were left untreated (**A**, lanes 1–4) or treated (**C**, lanes 1–4) with 20 μM PP2 for 12 h. Later, cells in A and C were left either un-infected (MOCK) or HSV-1 infected in the absence **(A)** or presence **(C)** of 20 μM PP2. After 4, 8, and 18 h post-infection (hpi) 20 μg of cell extracts were subjected to SDS-PAGE followed by Western blotting. **(A,C)** Show immunodetection of Src phosphorylated at residue Y424 (p-Src), total Src (Src), Dyn 2 phosphorylated at residue Y231 (p-Dyn2), and total Dyn2 (Dyn2). Western blotting with antibodies to ICP8 and β-tubulin were used to control HSV-1 viral expression and loading control, respectively. **(B,D)** Show normalized values of active forms of Src and Dyn2 proteins obtained after densitometric quantification of the blots expressed as the ratio of the phosphorylated/total Src or phosphorylated/total Dyn2 proteins. Bars represent the mean ± *SD* of three independent experiments. ^***^p < 0.001, ^**^p < 0.01, ^*^p < 0.05; ns: non-significant compare to the MOCK. Brackets display treatment comparisons.

**Figure 2:**
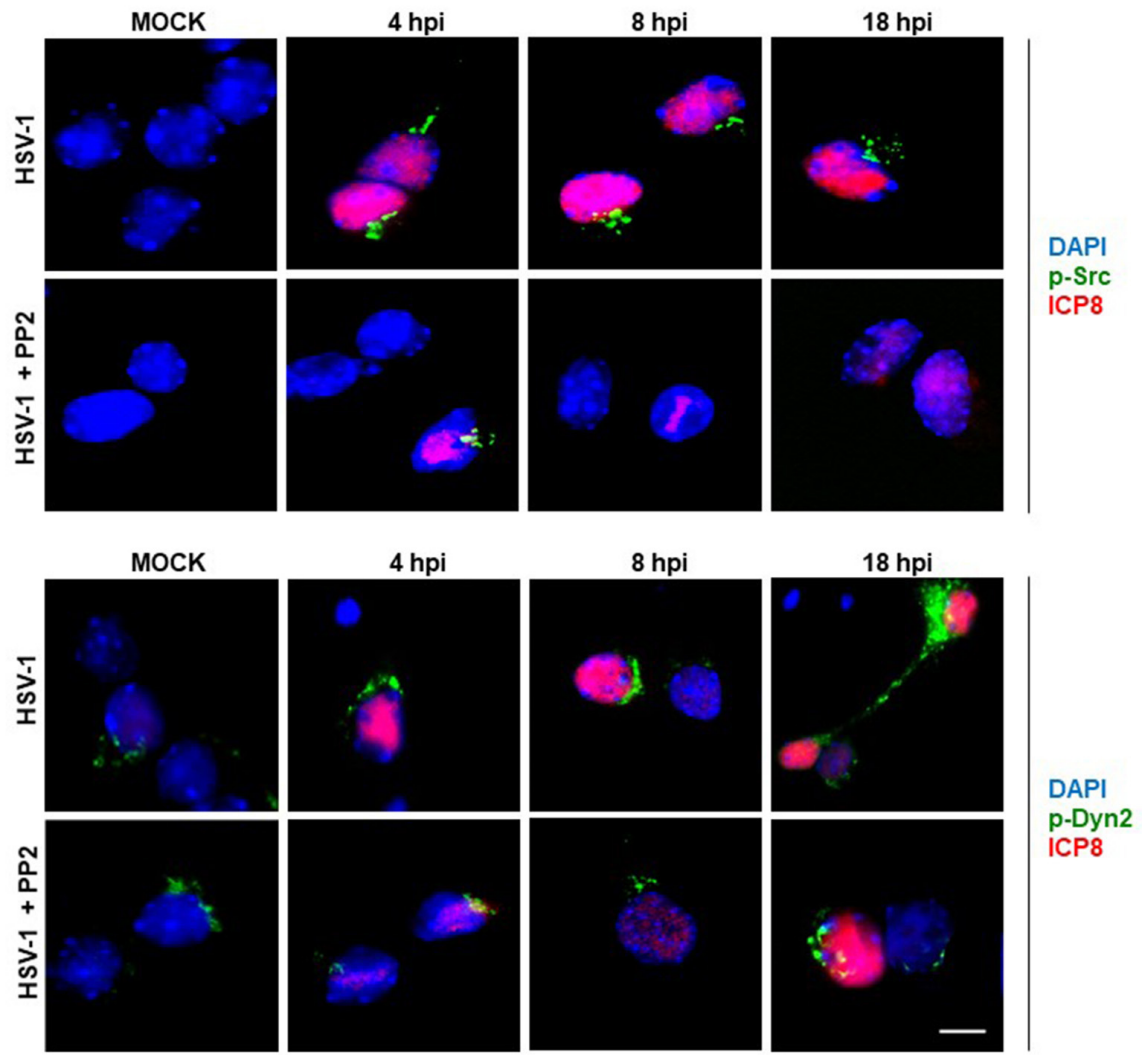
Localization of Src kinase and Dyn2 during HSV-1 neuronal infection and Src kinase inhibition. Cortical primary neurons were left untreated (HSV-1) or treated (HSV-1 + PP2) with 20 μM PP2 for 12 h, and then, cells were either mock-infected (MOCK) or HSV-1 infected (moi = 10). After 4, 8, and 18 h post-infection (hpi) cells were fixed, permeabilized, and incubated with the rabbit polyclonal antibodies to either p-Src **(Upper)** or p-Dyn2 **(Lower)** and with the mouse monoclonal antibody to ICP8 (both panels). Next, cells were incubated Alexa Fluor 488–conjugated donkey anti-rabbit IgG (green channels) and Alexa Fluor 594–conjugated donkey anti-mouse IgG (red channels). DAPI stain was used to visualize nuclei. Images were acquired by fluorescence microscopy Merging green and red channels generated a third image show in each row. Scale bar, 5μm. For visualization of neuronal morphology see also Figure S1.

In the present study we evaluated if HSV-1 could activate Src tyrosine kinase in cortical primary neurons to this end, we studied the relative levels of phosphorylated Src-Tyr424, a highly conserved phosphorylation site among all SFKs members, crucial modification for Src kinase activation (Roskoski, 2005; Ingley, 2008). One known substrate of Src tyrosine kinase is Dyn2, which is phosphorylated at Tyr231, residue located in the activation loop of its GTPase domain that under phosphorylation triggers its GTPase activity (Ahn et al., 1999; Ahn, 2002; Shajahan, 2004). As shown in Figures 1A,B levels of phosphorylated Src-Tyr424 (p-Src) and phosphorylated Dyn2-Tyr231 (p-Dyn2) were significantly increased during early HSV-1 infection (4 hpi) of neurons, reaching maximal values at 18 hpi, and compared with un-infected neurons (MOCK). To control specific activation of Src tyrosine kinase by HSV-1 infection we treated cells with the selective Src kinase inhibitor named PP2 (20 μM) (Figure 1B). We observed that treatment with PP2 caused a significant reduction in the phosphorylated levels of p-Src and p-Dyn2 (≥93 and 44%, respectively) after 8 hpi (Figures 1C,D). Confocal immunofluorescence analysis confirmed these results observing a notorious increase of p-Src and p-Dyn2 in HSV-1 infected neurons compared to MOCK cells (Figure 2). Moreover, we observed that both, p-Src and p-Dyn2, distributed into a perinuclear region in infected neurons. Interestingly, the expression of viral protein ICP8 was delayed by the treatment with PP2, suggesting that HSV-1 replication and late viral expression could also be affected by specific Src tyrosine kinase inhibition (Figure 2 and Figure S2).

### Golgi disruption during HSV-1 neuronal infection

**Figure 3:**
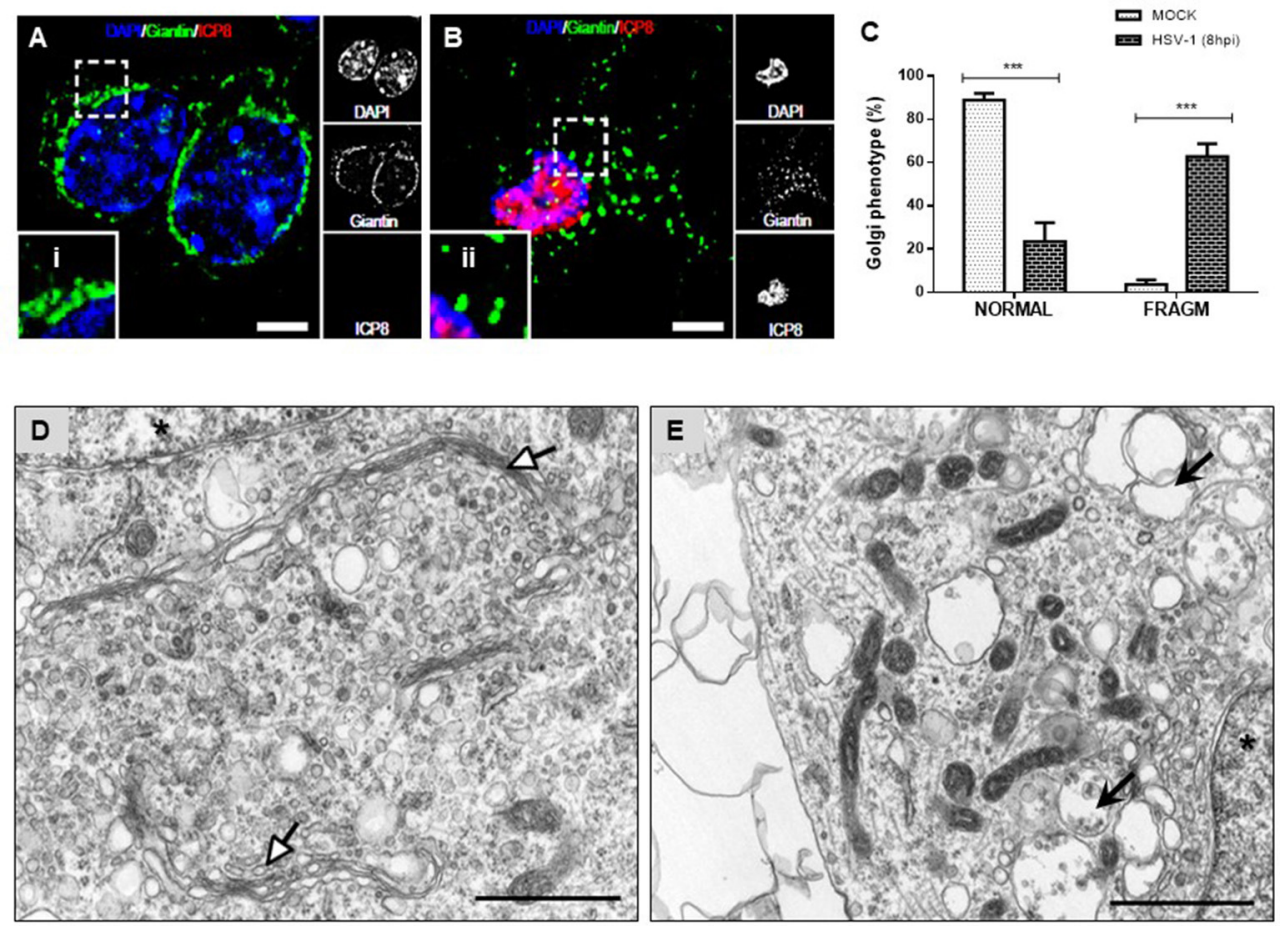
Morphological disruption of the GA during HSV-1 neuronal infection. Cortical primary neurons were either mock-infected (**A**; MOCK) or HSV-1 infected **(B)**. After 8 h post-infection cells were fixed, permeabilized, and immunostained for Giantin (Golgi marker protein, green) and ICP8 (viral infection marker, red). Nuclei were visualized with DAPI. **(A,B)** Show the corresponding merged images. Regions of interest taken from the merged images (left box) are presented at high magnification to show (i) normal distribution of Giantin marker (concentrated in the perinuclear region) and (ii) fragmented distribution (dispersed throughout the cytoplasm). Scale bar, 5 μm. **(C)** Scoring of Golgi morphologies (distribution pattern of Giantin) in mock- and HSV-1-infected neurons at 8 hpi. Golgi phenotypes in these cells were categorized as normal reticular-perinuclear signal (NORMAL) or displaying fragmentation/vesiculation of the Golgi compartment (FRAGM). Neuronal morphology was visualized with the specific neurites marker MAP-2 as shown in Figure S1. Bars represent the mean ± *SD* of three independent experiments. ^***^p < 0.001 compare to the MOCK. Brackets display a comparison between infected and mock-infected neurons. Representative electron micrographs of Golgi stacks in mock-infected neurons **(D)** and in HSV-1 infected neurons at 8 hpi **(E)**. Organized Golgi stacks are indicated in mock-infected neurons (white arrowheads), structures that are absent in HSV-1-infected cells, which displayed discontinued stacks and dilated-elongated Golgi cisternae (black arrowheads). Asterisk indicate nucleus. Scale bar, 1 μm.

Because previous studies demonstrated that GA integrity is regulated by Src tyrosine kinase activity we assessed whether neuronal HSV-1 infection could trigger changes at the GA. Figure 3 shows that during the course of neuronal HSV-1 infection (8 hpi) the GA pattern is drastically altered, compared to MOCK cells. We observed that Giantin, a conserved protein of the GA, shows a scattered and fragmented distribution through the cytoplasm, compared to the highly ordered, pericentriolar, ribbon-like structure distribution observed in mock cells (Figure 3B, compared to Figure 3A). This redistribution caused no difference in Giantin protein levels during HSV-1 neuronal infection (data not shown). Infected neurons were identified by the nuclear positive detection of the viral protein ICP8 by immunofluorescence. A similar fragmented pattern was observed with the *cis*-Golgi matrix protein, GM130 (Figure S3). Quantification analysis of this phenotype showed that over 60% of HSV-1 infected neurons (8 hpi) exhibited a fragmented Golgi phenotype (FRAGM) (Figure 3C). As a control, we determined that the increase in FRAGM was not the result of apoptosis activation (Figure S4). Importantly, these morphological alterations of the GA were observed only in infected neurons that were positive to ICP8 viral marker, demonstrating that the effect is specifically caused by productive infection (Figure 3B and Figure S4). Further, we characterized the ultrastructural features of the GA upon HSV-1 productive infection. Transmission electron microscopy revealed swollen cisternae and disorganized stacks in HSV-1 infected neurons (8 hpi) (Figure 3E), in contrast to the expected highly organized Golgi stacks in MOCK cells (Figure 3D).

### Fragmentation of the GA by HSV-1 neuronal infection is dependent on Src tyrosine kinase activity

**Figure 4:**
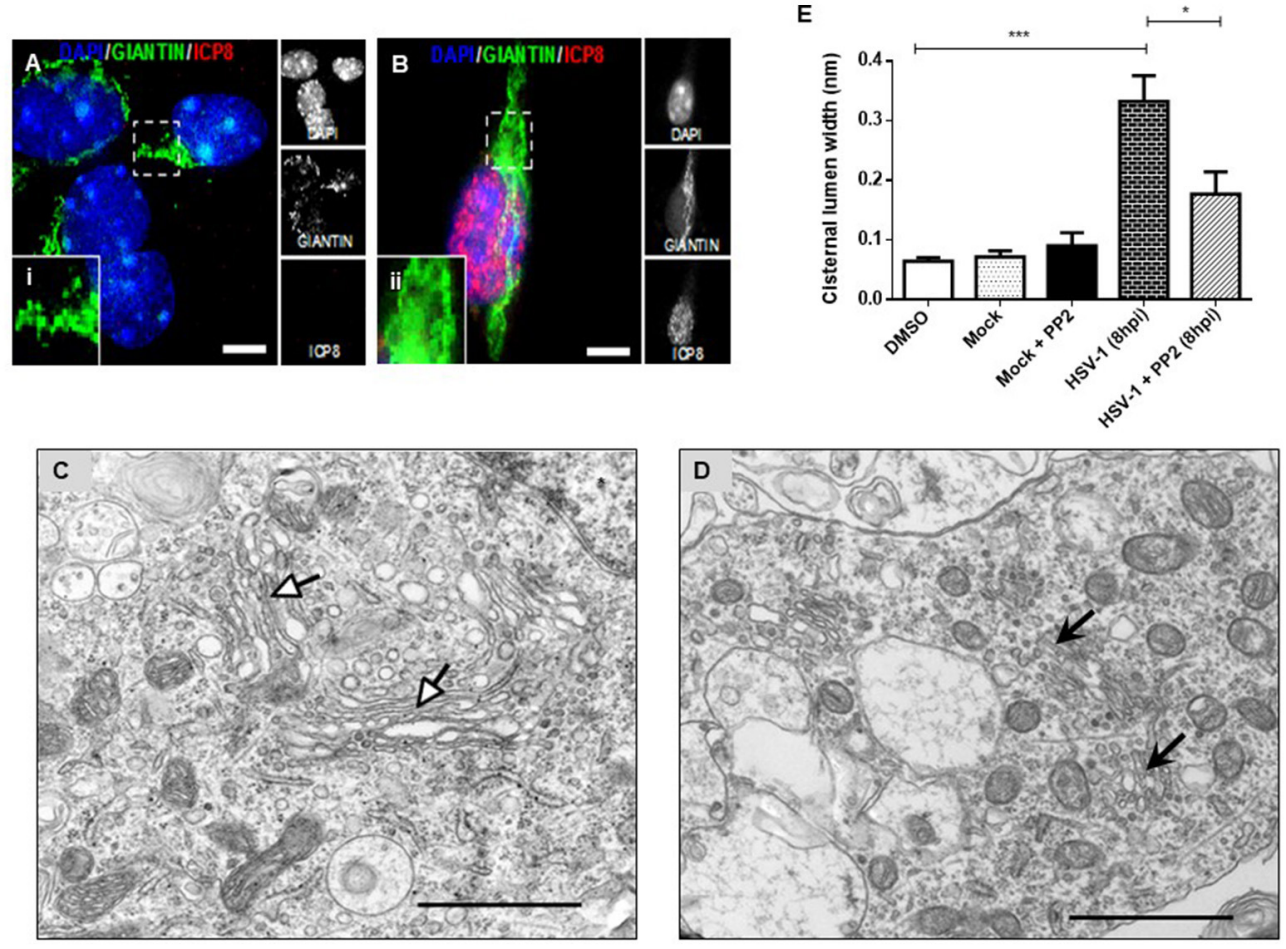
PP2, a specific Src kinase inhibitor, reduced Golgi apparatus alterations observed during HSV-1 neuronal infection. Cortical primary neurons were treated with 20 μM PP2 for 12 h, then cells were left either mock-infected (**A**; MOCK) or HSV-1- infected **(B)** during 8 h in the presence of the inhibitor. After, cells were fixed, permeabilized, and immunostained for Giantin (Golgi marker, green) and ICP8 (viral infection marker, red). Nuclei were visualized with DAPI. **(A,B)** Show the corresponding merged images. **(A,B)** Show boxes (i–ii) with higher magnification indicating normal distribution of Giantin. Scale bar, 5 μm. Representative electron micrographs of Golgi stacks in mock-infected neurons **(C)** and in HSV-1 infected neurons at 8 hpi **(D)**, both treated in the presence of PP2. Black arrowheads indicate that in the presence of PP2 there is a consistent reduction of the disrupted Golgi cisternae flatness and morphology trigger by HSV-1, observing similar results than in MOCK cells (white arrowheads). Asterisk indicated nucleus. Scale bar, 1 μm. **(E)** Quantitation of Golgi cisternae lumen (μm) in mock- and HSV-1- infected cells in the absence or presence of 20 μM PP2 for 12 h. In addition, treatment with 20 μM PP2 alone and DMSO vehicle control was also included. Data represents the average maximum luminal width of Golgi cisternae. Bars represent the mean ± *SD* of three independent experiments. ^***^p < 0.001, ^*^p < 0.05. Brackets display treatment comparisons.

To determine whether fragmentation of the GA triggered by HSV-1 infection was dependent on neuronal Src tyrosine kinase activity, primary cortical neurons were infected with HSV-1 in the absence or presence of PP2 inhibitor, a selective inhibitor of Src-family kinases. Immunofluorescence analyses showed that Giantin distribution in cells infected with HSV-1 in the presence of PP2 (Figure 4B) was almost similar to uninfected cells (Figure 4A). Further, to investigate whether inhibition of Src tyrosine kinase had a most profound effect in the GA morphology, we performed an ultrastructural characterization of the effect of PP2 upon HSV-1 infection. We observed that HSV-1 infected neurons treated with PP2 showed intact and tightly compacted Golgi structures displaying few distended cisterna (Figure 4D). Quantitation of the maximum luminal width of the Golgi cisternae (μm) showed a significant increase in this measurement in HSV-1 infected neurons compared to controls. Importantly, this parameter was significantly reduced after PP2 treatment (Figure 4E). These results strongly suggest that Src tyrosine kinase activity is required to trigger Golgi fragmentation in HSV-1 infected neurons.

### Activation of Dyn2 and fragmentation of the GA by HSV-1 requires VP11/12 in neuronal cells

**Figure 5:**
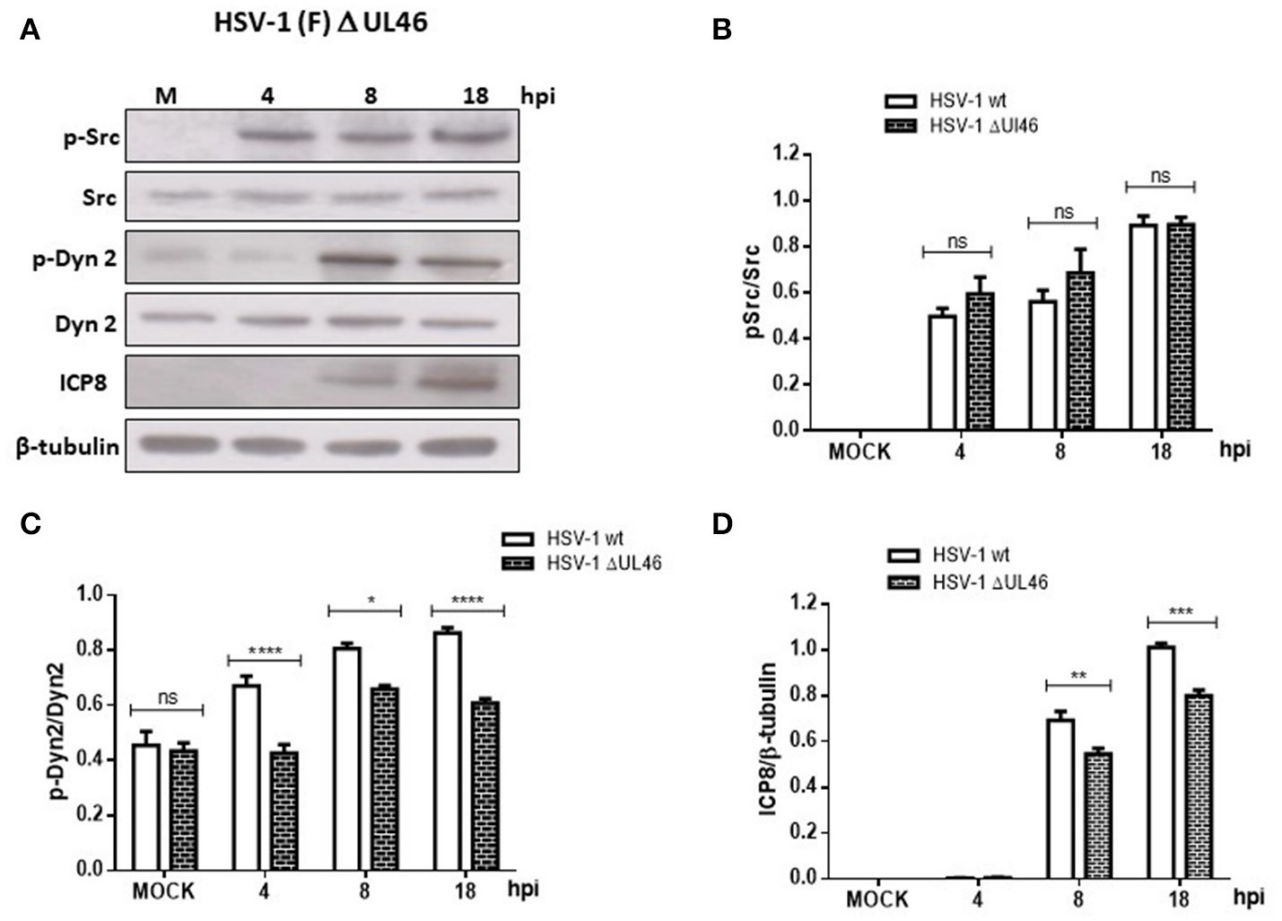
Neuronal response to HSV-1 infection lacking the HSV-1 tegument protein VP11/12. **(A)** Cortical primary neurons were infected with either HSV-1 wild type or a deleted HSV-1 version that lacks the viral gene UL46 (HSV-1 ΔUL46). After 4, 8, and 18 h post-infection (hpi) equivalent amounts of cell extracts were subjected to SDS-PAGE followed by Western blotting and normalized levels of p-Src/Src **(B)**, p-Dyn2/Dyn2 **(C)**, and ICP8/β-tubulin **(D)** determined. Bars represent the mean ± *SD* of three independent experiments. ^****^p < 0.0001, ^***^p < 0.001, ^**^p < 0.01, ^*^p < 0.05, Brackets display treatment comparisons.

**Figure 6:**
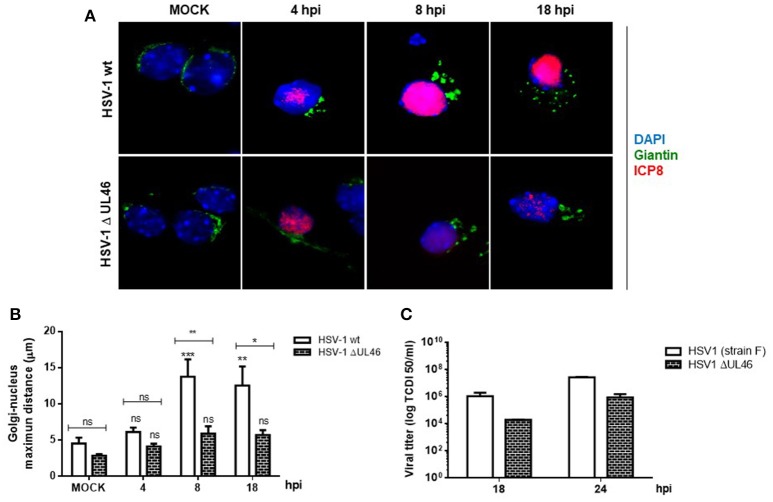
VP11/12 tegument protein is involved in the GA fragmentation triggered by HSV1 infection in neurons. **(A)** Cortical primary neurons were mock-infected (MOCK) or either infected with wild type HSV-1 or a deleted HSV-1 version that lacks the viral gene UL46 (HSV-1 ΔUL46) coding for VP11/12 protein. After 4, 8, and 18 h post-infection (hpi) cells were fixed, permeabilized, and immunostained for Giantin (Golgi marker protein, green) and ICP8 (viral infection marker, red). Merging green and red channels generated the third image show in each row. Nuclei were visualized with DAPI. Scale bar, 5 μm. **(B)** Quantitation of the average distance of Giantin positive structures to the nucleus (μm) under the conditions described in **(A)**. Bars represent the mean ± *SD* of three independent experiments. ^***^p < 0.001, ^**^p < 0.01, ^*^p < 0.05; ns, non-significant compare to the MOCK. Brackets display treatment comparisons. **(C)** Neuronal cultures were harvested at these times and the production of infectious viral progeny was measured in the supernatants by a standard TDCI 50 assay.

Because previous studies in T cells have demonstrated that HSV-1 tegument protein VP11/12 (viral gene UL46) triggers Lck activation, a member of the SFK family (Wagner and Smiley, 2009), we hypothesized that VP11/12 could be crucial for the activation of Src tyrosine kinase. To test this hypothesis, we characterized the effect of a deleted HSV-1 version that lacks the viral gene UL46 (HSV-1ΔUL46), comparing this version to the wild type. First, normalized data from Western blotting analysis showed an indistinguishable response regarding the levels of phosphorylated Src-Tyr424 between these two HSV-1 versions, strongly suggesting that VP11/12 is dispensable for the activation of Src tyrosine kinase (Figures 5A,B). Next, we analyzed the relative levels of p-Dyn2 (Figures 5A,C). We showed that cells infected with HSV-1 ΔUL46 had a significant decrease in Dyn2 activation, compared to cells infected with HSV-1 wild type (Figure 5C). Further, we found that lack in VP11/12 caused a significant reduction in the expression of the viral ICP8 protein compared to the wild type version (Figure 5D and Figure S2) suggesting that HSV-1 lacking VP11/12 replicates less efficiently. Finally, to investigate whether VP11/12 could play a role in GA fragmentation we studied the distribution of Giantin in primary cortical neurons infected with HSV-1 ΔUL46. We observed that lack in VP11/12 reduced the scattered and fragmented distribution of Giantin observed with the HSV-1 wild-type version (Figure 6A). Quantification analysis of the distance of Giantin positive structures to the nucleus showed a significant reduction in this parameter at 8 and 18 hpi (Figure 6B), however in these cells, viral replication was significantly reduced in comparison with those infected with the wild type virus (Figure 6C). Additionally, VP11/12-GFP accumulated in discrete puncta throughout the cytoplasm without affecting the normal localization of Dyn2, Giantin and Src (Figure S5). Furthermore, Src phosphorylated at Tyr424 was undetectable either in cells expressing VP11/12-GFP or transfected with the empty vector (Figure S5). Altogether, these results indicate that HSV-1 tegument protein VP11/12 is dispensable for the activation of Src tyrosine kinase, and is necessary but not sufficient to induce Dyn2 activation and GA fragmentation.

## Discussion

In neurons, neurotransmitters and proteins that are released during synaptic activity are manufactured by the endoplasmic reticulum and the GA (Bauerfeind and Huttner, 1993). Proteins are continuously transported from the GA to synapses along the axon (Bauerfeind and Huttner, 1993), however how HSV-1 reactivation perturbs these processes is unknown. Previous evidence had demonstrated that HSV-1 can modulate intracellular signaling pathways that are normally involved in cellular homeostasis and neuronal integrity (Benetti and Roizman, 2006, 2007; Peri et al., 2008; Walters et al., 2010; Hafezi et al., 2012; Boutell and Everett, 2013; Sen et al., 2013), however the impact of the regulation of these signaling pathways in the overall maintenance of the organelles in neurons has been poorly characterized.

A series of discoveries have determined that neurodegenerative pathologies such as Alzheimer's disease, Amyotrophic Lateral Sclerosis, Huntington's, and Parkinson (Sakurai et al., 2000; Sun et al., 2008; Joshi and Wang, 2015) present early structural alterations of the GA, that affect intracellular signaling pathways associated with the maintenance of specific neuronal functions and phenotype identity (Bossy-Wetzel et al., 2004; Liang et al., 2008; Imtiaz et al., 2014; Joshi and Wang, 2015). However, the molecular basis for Golgi fragmentation and its effects on disease progression have remained largely unexplored. Diverse studies have previously shown that HSV-1 uses the secretory pathway of epithelial cells, affecting the functional organization of the GA in these cells (Campadelli et al., 1993; Avitabile et al., 1995).

In the same context, a previous study showed that expression of a constitutively active form of Src tyrosine kinase resulted in GA fragmentation, which depends on Dyn2 activity (Weller et al., 2010). Furthermore, Dyn2 has also been shown to participate in membrane trafficking pathways including endocytosis playing a role in membrane fusion and in the regulation of microtubule and actin cytoskeleton dynamics (González-Jamett et al., 2014). However, how Dyn2 controls infectivity and productive viral infection is not yet known. Here we demonstrated that HSV-1 productive infection triggers activation of Src and Dyn2, however the precise step that is regulated by this GTPase during HSV-1 viral infection is still a mystery.

**Figure 7:**
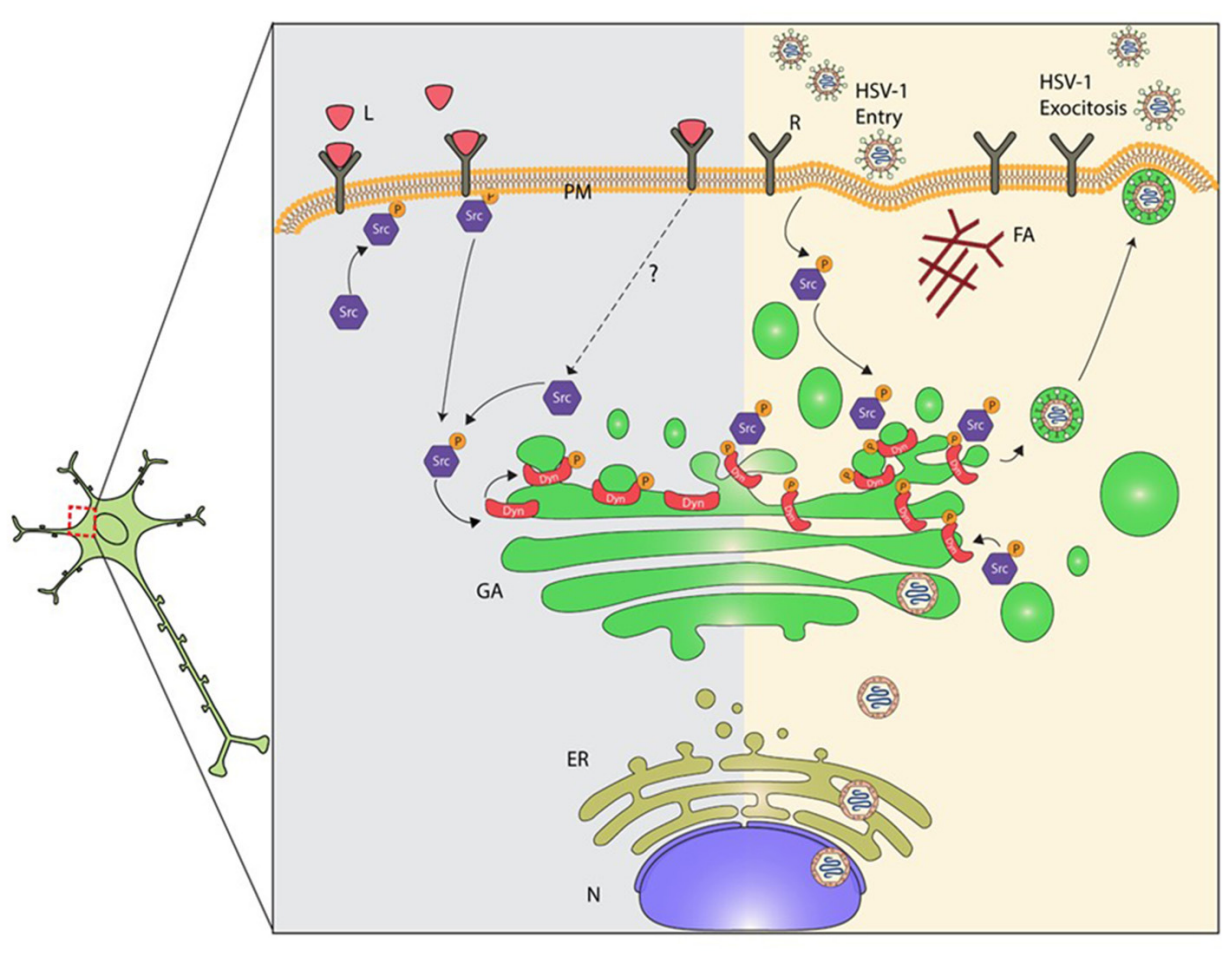
Proposed model for mechanisms underlying dysruption of the GA during HSV-1 neuronal infection. Alteration of the normal neuronal GA morphology by HSV-1 involves the activation of Src kinase signaling induced by the interaction of HSV-1 with cellular receptor(s). The prolonged activation of Src kinase activity during HSV-1 early infection triggers phosphorylation of Dyn2 (Tyr231), enhancement of Dyn2 assembly and GTPase activity at the GA. High Dyn2 activity induces membrane vesiculation, affecting GA morphology and function. In addition, assembly of HSV-1 virions ends with the acquisition of the viral envelope at the *cis*-Golgi, followed by their release from vesicles that emerge from the *trans*-Golgi network. We propose that activation of Src kinase and Dyn2 at the GA perturbs Golgi integrity affecting the secretory pathway in neurons. Consequently, alteration of the normal secretory pathway could impair and compromise neuronal function, and thereby contribute to neurodegenerative disorders development. L, ligands; R, cellular receptor; PM, plasma membrane; AF, actin filament; Src, Src kinase; P, phosphorylated; Dyn2, Dynamin 2 protein; G, Golgi; ER, endoplasmic reticulum; N, nucleus.

Here we show that infection of primary neurons with HSV-1 triggers activation of Src tyrosine kinase and its downstream target Dyn2, leading to GA fragmentation (Figure 7). Activation of Src was evidenced by increased phosphorylation of the conserved tyrosine residue (Tyr 424), which lies in the activation loop of the kinase domain. Phosphorylation of this site is present in the most highly active forms of SFKs and it appears to be the most important phosphorylation site for maximal kinase activity (Ingley, 2008). One possible explanation by which HSV-1 could trigger activation of Src is the direct binding of the virus to cellular receptors. Most herpesviruses use integrins as key components for cell entry (Campadelli-Fiume et al., 2007). One example is the interaction of virion gH/gL glycoproteins with αvβ8-integrin receptor, which is located at lipid microdomains and requires Dyn2 for viral entry (Gianni et al., 2010). Similarly, the interaction of Kaposi's sarcoma associated herpes virus (KSHV) gB protein with host cell surface receptors activates the host integrin-dependent focal adhesion kinase (FAK), that leads to binding of the SH2 domain of Src family kinases (Veettil et al., 2014). Interestingly, Wang et al. (2011) demonstrated that Dyn2 is recruited to focal adhesions (FAs) by a direct interaction with FAK, and Src–mediated activation of Dyn2 promotes FA turnover by inducing the endocytosis of integrins. Another possible mechanism is the one based on the binding of HSV-1 to the epidermal growth factor receptor (EGFR), which was previously shown to be quickly and transiently activated and clustered when the virus was added to neuronal cells (Zheng et al., 2014). In this context, SFK have been shown to be necessary for signaling from EGFR to PI3K (Reddy et al., 2016). Moreover, EGFR is involved in activation of SFKs and other cellular tyrosine kinases adjacent to the membrane, promoting the formation of an intracellular signaling platform, responsible for regulating the cellular cytoskeleton and adequate maintenance of neuronal function, a strategy that allows maturation, trafficking and egress of a new viral progeny from infected cells (Zheng et al., 2014).

Alternatively, once the virus enters the cell, viral proteins could interact directly with SFKs, resulting in Src activation and subsequent phosphorylation of viral proteins (Wagner and Smiley, 2011). This is the case for the tegument protein VP11/12, which utilizes tyrosine-based motifs within its C-terminal region to bind the SH2 domains of Src kinases family members, in particular, Lck, stimulating the PI3K/Akt pathway by mimicking an activated growth factor receptor in T cells (Strunk et al., 2016). Concerning this last mechanism, here we show that VP11/12 is not critical for Src and Dyn2 activation in neurons, but surprisingly partially contributes to GA fragmentation, suggesting that in addition to VP11/12, HSV-1 could use other viral proteins, to articulate Src and Dyn2 signaling, involved in the changes in the GA triggered by viral infection.

Regardless the actual mechanism(s) used by the virus to activate Src, our results show that Src activation leads to an increased phosphorylation/activation status of the GTPase Dyn2. In fact, phosphorylation of Dyn2 was correlated with Src activation and Golgi fragmentation during the course of infection (Figure 7). Although different tyrosine kinases could phosphorylate Dyn2, the fact that treatment with PP2, previous to HSV-1 infection, reduced the levels of p-Dyn2 strongly suggest the involvement of Src or a closed related member of the SFKs family. Concerning the subcellular localization described for Src, Chu et al. (2014) showed that before activation, Src is concentrated on one side of the perinuclear compartment associated to the GA. This localization is important for its ability to induce polarized movement to the cell periphery shortly after kinase activation. Intriguingly, we observed that p-Src is mainly distributed in the perinuclear region during the course of HSV-1 infection (4–18 hpi). Because intracellular distribution of Src tyrosine kinase depends on intact microtubules (Arnette et al., 2016), it is plausible that concentration of p-Src to the perinuclear region could be the result of microtubule instability along the axons, due to the cleavage, hyperphosphorylation, and concentration of tau protein by HSV-1 (Zambrano et al., 2008) or the activation of Dyn2 (Ishida et al., 2011). On the other hand, it has been reported that direct association of Dyn2 with cortactin, an actin-binding protein, reduces association of Dyn2 to the GA (Cao et al., 2005). Therefore, concentration of active Dyn2 at the perinuclear region during HSV-1 infection could be also a consequence of an altered interaction of p-Dyn2 with cortactin. In this context, it is tempting to speculate that HSV-1 favors sequestration of activated versions of Src and Dyn2 at the perinuclear region to facilitate assembly and progeny egress process. The assembly of HSV-1 virions ends with the acquisition of the viral envelope proteins at the *cis*-Golgi, followed by their release from vesicles that emerge from the TGN (Avitabile et al., 1995). Our findings suggest that activation of Src tyrosine kinase and Dyn2 by HSV-1 infection regulates Golgi integrity and vesiculation during the secretory process.

Our results showed that HSV-1 triggers GA fragmentation in neurons. In agreement with these findings, previous studies have demonstrated that HSV-1 infection induces a similar phenotype in epithelial cells, which has been confirmed by electron microscopy (Campadelli et al., 1993). Moreover, we showed that this phenotype was rescued by PP2.

Ultrastructural analysis showed that the average maximum cisternal width (0.194 μm) of HSV-1 infected neurons treated with PP2, was similar to the width determined in mock neurons treated with PP2 (Figure 4). Thus, the results obtained are consistent with the report of Weller et al. (2010) showing that the transit of a viral protein (G protein) of the Stomatitis vesicular virus in epithelial cells generates substantial activation of the Src kinase, phosphorylation of dynamin 2 at the tyrosine residue (Y231), and GA alteration. In addition, this phenomenon was prevented by pretreating cell cultures with PP2 or by expressing a non-phosphorylable mutant of Dyn2 (Dyn2YF), or deficient in its activation (Dyn2K44A).

On the other hand, Liang et al. (2008) and Liang and Roizman (2006) suggested that SFKs modulation during epithelial infection might be dependent on HSV-1 viral proteins such as ICP0, where physical interaction between ICP0 and kinases occurs through sequences between residues 245–510 of ICP0 and SFKs SH3 domain. Likewise, the viral protein VP11/12 also interacts with SFKs members (LcK) in lymphoid cells, potentiating its own post-translational modifications and activation of LcK (Wang et al., 2011). VP11/12 is one of the most abundant HSV-1 tegumental proteins, having a role as a structural component of the mature virion and as a modulator of cellular and/or viral functions on infected cells. Its participation was identified in cellular signaling pathways such as PI3K-Akt (Wagner and Smiley, 2011; Strunk et al., 2013) and it has the ability to associate to the plasma membrane of HSV-1 infected cells (Willard, 2002; Murphy et al., 2008).

These results contribute with new evidence regarding the possible role of the Src kinase dependent signaling pathway on the structure and function of neuronal GA during HSV-1 infection. Interestingly, our findings suggest that the structural alteration of the GA observed during neuronal infection is at least partially dependent on the signaling platform linked to Src kinase, because inhibiting its activity during infection attenuated significantly the changes observed at the GA level. Evidence suggests that the alteration of GA in neurons is an early and probably irreversible event that could potentiate the neurodegenerative process, but not a secondary consequence of the apoptotic process (Gonatas et al., 2006). However, despite the relevance of these findings, few previous reports have contributed with insights about possible candidates that could explain GA disturbances reported in neurons. Therefore, our results are the first to link HSV-1 infection with activation of the Src pathway and alterations of the GA in neurons, disturbances that can be responsible for the deleterious effects of HSV-1 in central nervous system in humans.

## Ethics statement

We used 20 pregnant mice, (considering minimal use of animal to validate results), provided by the Unit of Mice Breeding at the Department of Immunology. In this Unit animals are kept under standard conditions (temperature, feeding, light, and water). Trained personnel sacrificed mice using lethal doses of intravenous sodium pentabarbitone (200 mg/kg of total weight). Death was confirmed observing cessation of heartbeat and respiration, and absence of reflexes, in agreement with international standards (http://www.lal.org.uk). Our laboratories have the necessary suitable procedures for biological elements and chemicals disposal, according to the Safety Manual of Procedures and Handling of Wastes from the Universidad Austral de Chile, and Bio-safety Regulations from CONICYT. Animal handling was done according to the Experimentation Animals Regulations from the Universidad Austral de Chile, the protocol was approved by the Bioethical Committee of the Austral University of Chile. All the activities were supervised and authorized by the principal investigator.

## Author contributions

Conceived and designed the experiments: MC and CO. Performed the experiments: CM, MH, LL, YA, and CS. Analyzed the data: MM, PB, FC, MC, and CO. Contributed reagents/materials/analysis tools: MM, PB, FC, and CO. Wrote the paper: MC, PB, and CO. All authors have read and approved the final manuscript.

### Conflict of interest statement

The authors declare that the research was conducted in the absence of any commercial or financial relationships that could be construed as a potential conflict of interest.
