# Supplementary figures and images for: Herpes Simplex Virus Type 1 Neuronal Infection Perturbs Golgi Apparatus Integrity through Activation of Src Tyrosine Kinase and Dyn-2 GTPase
Source: Front Cell Infect Microbiol. 2017 Aug 22;7:371. doi: 10.3389/fcimb.2017.00371 (PMC5572415; doi:10.3389/fcimb.2017.00371)

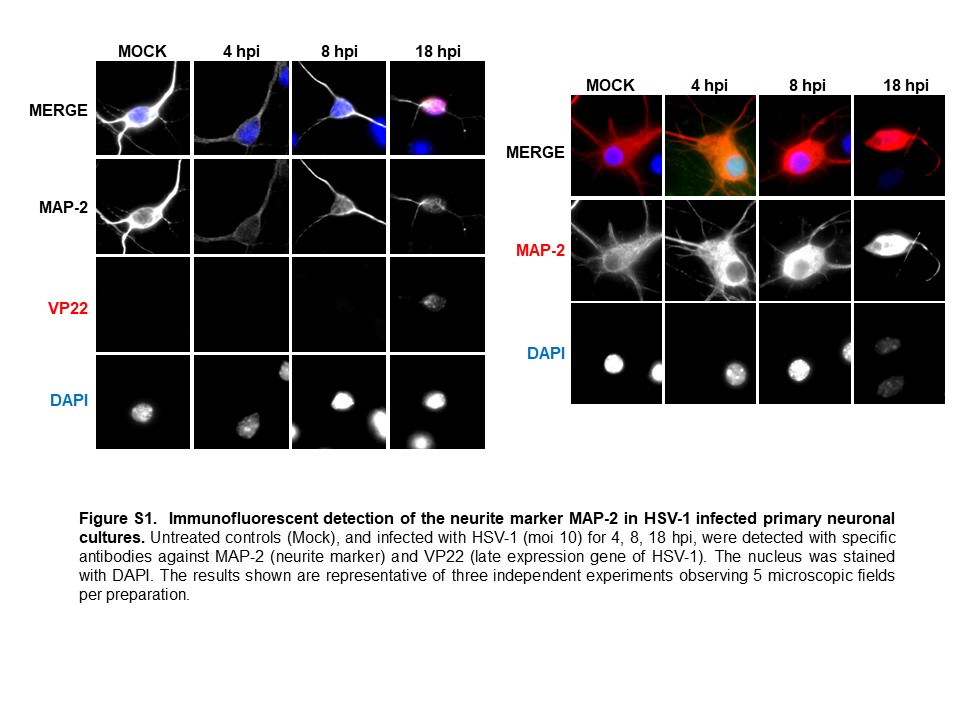

Supplement: Supplementary file 1 [file Image1.JPEG]

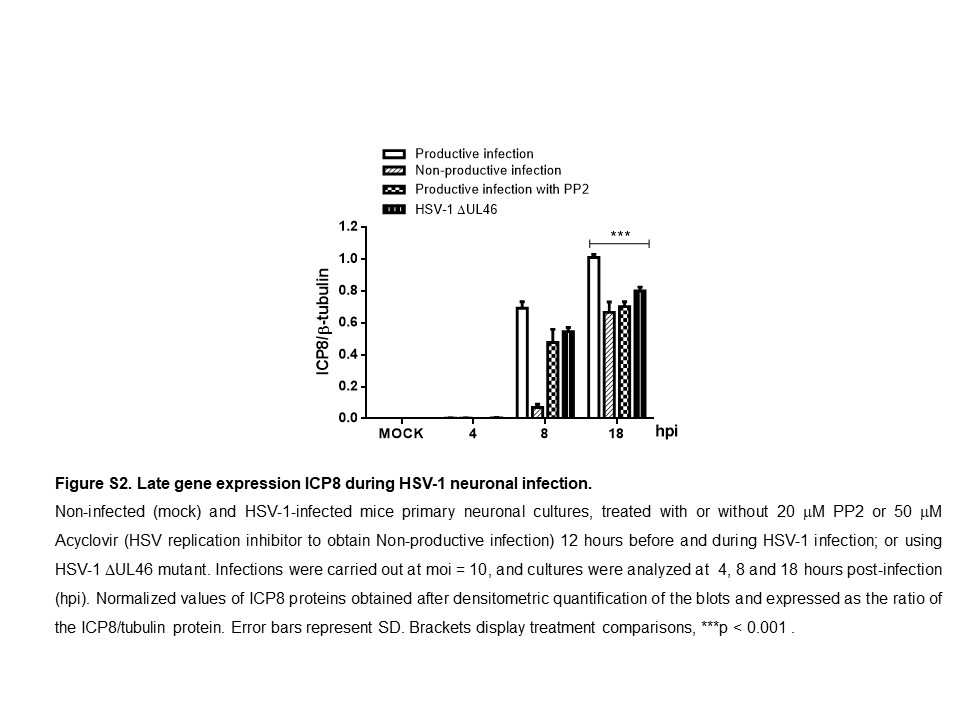

Supplement: Supplementary file 2 [file Image2.JPEG]

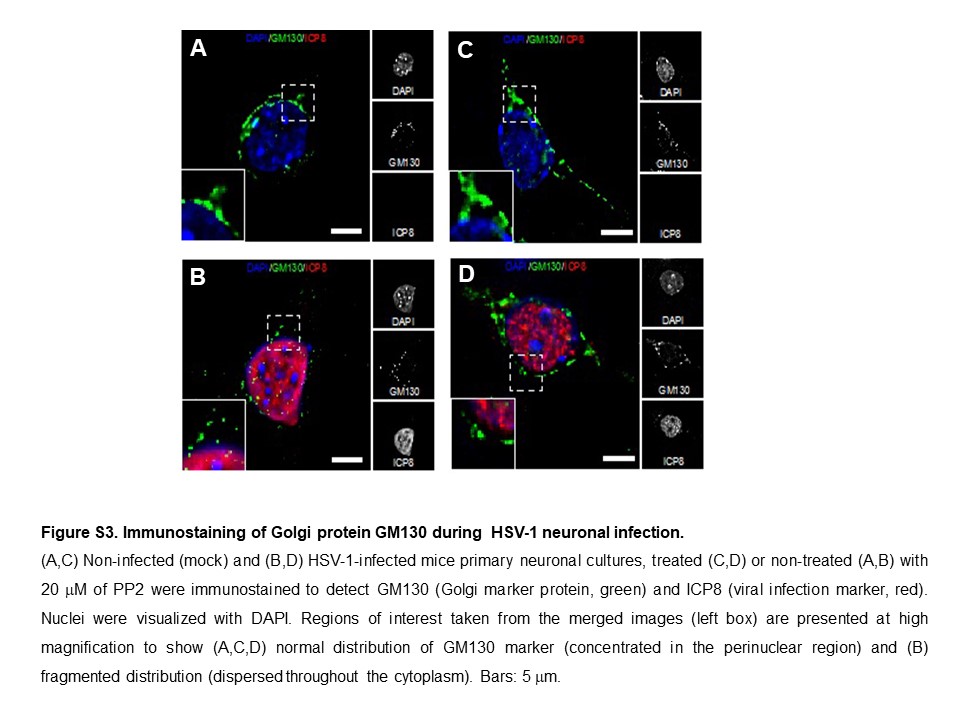

Supplement: Supplementary file 3 [file Image3.JPEG]

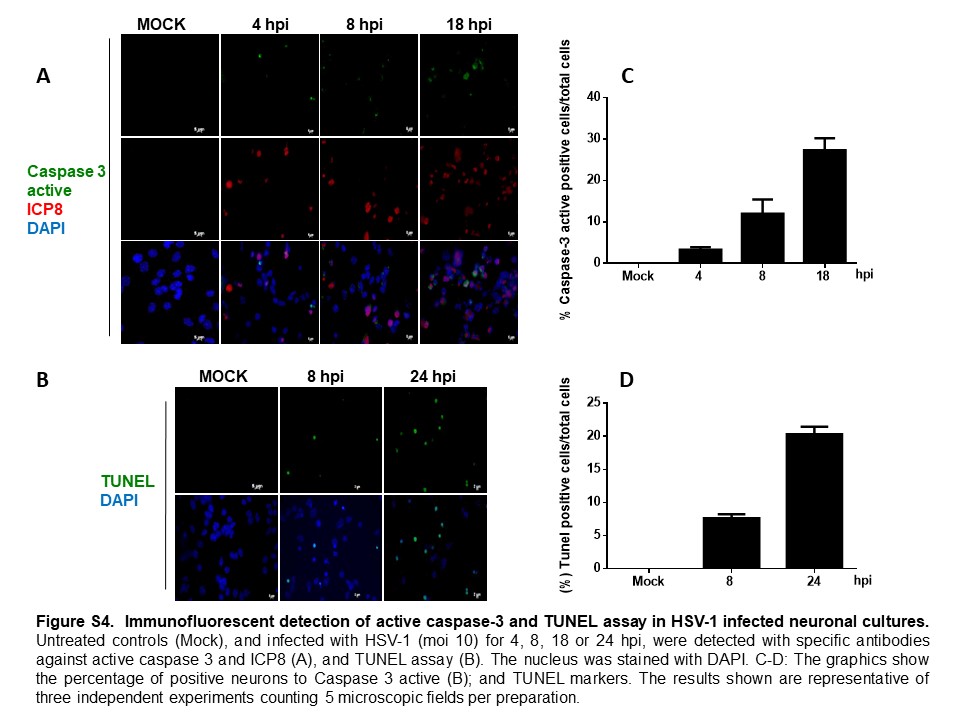

Supplement: Supplementary file 4 [file Image4.JPEG]

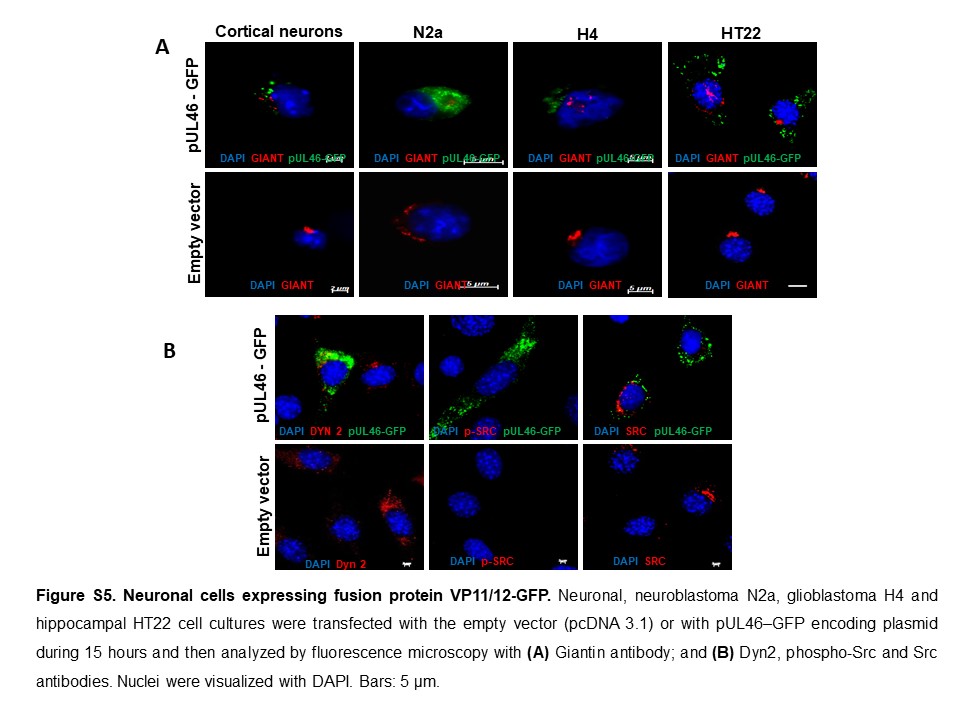

Supplement: Supplementary file 5 [file Image5.JPEG]
